# News and Coming Events

**Published:** 1907-12-28

**Authors:** 


### NEWS AND COMING EVENTS.

The Secretary of the Royal Society of Medicine notifies that the library and general offices of the Society will be closed from Monday, December 23, to December 30 next.

At the meeting of the Metropolitan Hospital Sunday Fund, to be held at the Mansion House on June 16, 1908, a bust of the late Mr. Geo. Herring will be formally unveiled.

The sum of £150,000 has been left to the city of Melbourne under the will of the late Mr. Edward Wilson. The gift is to be used "towards the erection of a new hospital for Melbourne."

Christmas at the Hospitals.—The children's Christmas-tree entertainment at the West End Hospital for Diseases of the Nervous System will be held at the hospital, 73 Welbeck Street, on Friday, December 27, at 3 p.m. At all the other hospitals the usual Christmas festivities will be held.

Presentations to Medical Men.—The medical men of Old Withington have presented Dr. Railton with a silver rose-bowl on the occasion of his retiring from the office of Medical Officer of Health of the district, which post he had held for thirty-five years. On his retirement from the Liverpool Royal Infirmary, Mr. Rushton Parker's students, past and present, are presenting him with his portrait in oils. Dr. William Berry has been presented with a silver tea-service in recognition of his twenty-five years' service as Honorary Secretary to the Wigan Medical Society.

West Ham and East London Hospital.—The annual dinner in aid of the funds of the institution was held on December 20 last at the Great Eastern Hotel. Alderman John Byford, jun., presided, and among those present were the Mayor of West Ham, Mr. T. Alexander Cook (chairman of the hospital), Canon Pelly, Mr. Masterman, M.P., Dr. C. J. Stocker, Dr. P. S. J. Nichol, the Rev. H. T. Spencer, and Mr. A. W. Scrivener (Secretary). The Chairman, in proposing "Success to the Hospital," urged that the work it had achieved justified, not only its maintenance, but a proposed extension which the Committee thought absolutely necessary. The in-patients treated last year numbered 568, of whom 348 were residents of West Ham, and 220 came from districts as far away as Leytonstone, Ilford, Hornchurch, Grays, Southend, and Woolwich. During the year there had been a considerable increase in local accidents. Owing to the lack of beds cases which would otherwise be detained sometimes had to be given first aid and then sent on to the London Hospital. The proposed extension would bring up the accommodation from sixty to one hundred beds, and the cost of maintenance would consequently be raised to nearly £4,000 per annum. In 1906 the out-patients' visits numbered 95,805, and for the current year that figure would be very much enhanced. The visitors for the King's Hospital Fund had approved the extension plans, and had expressed their opinion "that the hospital was very economically and efficiently worked in a very poor neighbourhood." As a result of their report the Council of the King's Hospital Fund had awarded the institution an addition of £250 to its grant.

